# Actions of Adenosine on Cullin Neddylation: Implications for Inflammatory Responses

**DOI:** 10.1016/j.csbj.2014.10.002

**Published:** 2014-10-15

**Authors:** Valerie F. Curtis, Stefan F. Ehrentraut, Sean P. Colgan

**Affiliations:** a Mucosal Inflammation Program and Department of Medicine, University of Colorado Anschutz Medical Campus, Aurora, CO 80045, United States; b Department of Anesthesiology, University of Bonn, Germany

**Keywords:** Inflammation, Nucleotide, Mucosa, Colitis, Epithelium, Murine model

## Abstract

There is intense interest in understanding how the purine nucleoside adenosine functions in health and during disease. In this review, we outline some of the evidence that implicates adenosine signaling as an important metabolic signature to promote inflammatory resolution. Studies derived from cultured cell systems, animal models and human patients have revealed that nucleotide metabolism is significant component of the overall inflammatory microenvironment. These studies have revealed a prominent role for the transcription factors NF-κB and hypoxia-inducible factor (HIF) and that these molecules are post-translationally regulated through similar components, namely the neddylation of cullins within the E3 ligase that are controlled through adenosine receptor signaling. Studies defining differences and similarities between these responses have taught us a number of important lessons about the complexity of the inflammatory response. A clearer definition of these pathways has provided new insight into disease pathogenesis and importantly, the potential for new therapeutic targets.

## 1 Introduction

Adenosine is a purine nucleoside that has long been implicated in normal cell growth and metabolism and more recently has been demonstrated to possess potent anti-inflammatory properties. Surprisingly little is known about the actual mechanisms of adenosine-mediated anti-inflammation. While signal transduction through the various adenosine receptors is reasonably well characterized, less is known about post-receptor signaling events and consequences of such signaling. One recently appreciated mechanism suggests that adenosine acts on the proteasomal degradation machinery that controls inflammation-associated transcription factors. This short review focuses on specific molecular pathways that have been identified downstream of adenosine signaling, including NF-κB and hypoxia inducible factor (HIF) pathways, and explores their role in inflammation, resolution and as potential new targets for therapy.

## 2 Adenosine and Inflammation

At sites of injury and inflammation, adenosine is released and plays an important role in regulating inflammatory responses and limiting inflammatory tissue destruction [1]. Increased levels of adenosine have been detected during both inflammation and hypoxia and in these conditions, adenosine is both anti-inflammatory and cytoprotective [2,3]. The adenosine pathway has multiple points of regulation including metabolism of purine nucleotides via CD39 (ectonucleoside triphosphate diphosphohydrolase 1, E-NTPDase1) and CD73 (ecto-5′-nucleotidase, Ecto5′NTase), signaling via adenosine A2A and A2B receptors, and transport via ENT1/2 (equilibrative nucleoside transporters 1/2) [4,5]. Therefore, multiple potential therapeutic targets of the adenosine pathway have been evaluated for various inflammatory diseases and ischemia reperfusion disorders [6,7].

**Fig. 1:**
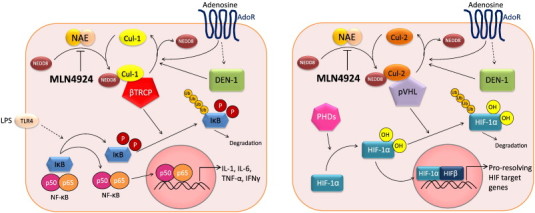
Neddylation pathways influencing NF-κB and HIF-1α. Left, NF-κB pathway: Pro-inflammatory stimuli, such as LPS, facilitate the phosphorylation of IκB, leading to the recognition of p-IκB by the Cul-1–Nedd8–βTRCP complex, culminating in its polyubiquitination and proteasomal degradation. The conjugation of Nedd8 to Cul-1 is required for polyubiquitination and is achieved through a multi-enzyme process wherein DEN-1 cleaves the Nedd8 precursor to its mature form, allowing for conjugation to cullin proteins. Loss of DEN-1, or pharmacological inhibition of Nedd8 conjugation by MLN4924 through inhibition of the Nedd8-activating enzyme (NAE), prevents the activation of Cul-1, preventing the liberation of NF-κB from IκB and quenching pro-inflammatory signaling. The binding of adenosine to adenosine receptors (AdoR) also results in deneddylation of Cul-1. Studies are ongoing regarding a potential regulation of DEN-1 activity by adenosine and AdoR. Right, HIF-1α pathway: In contrast to NF-κB, HIF-1α in its hydroxylated form is degraded by the proteasome after ubiquitination via the Cul-2–Nedd8–pVHL complex. Pharmacological inhibition of Cul-2 neddylation using MLN4924 stabilizes cellular HIF-1α levels, leading to increased transcription of pro-resolving HIF target genes. Loss of DEN-1 also positively influences barrier function of intestinal epithelial cells.

Recent work has defined the relationship between adenosine, adenosine receptor (AR) signaling, and anti-inflammation [4]. The signal transduction through the various ARs has been well characterized, however, less is known about the post-receptor events. Work in various cell types has shown that adenosine inhibits NF-κB activation through a number of distinct mechanisms, including elevation of intracellular cyclic adenosine monophosphate (cAMP) and activation of protein kinase A (PKA) which blocks IκB phosphorylation thus inhibiting NF-κB activation [8], inhibition of tumor necrosis factor (TNF)-α-induced NF-κB activity and subsequent gene expression by inhibition of nuclear translocation of active NF-κB without influencing IκB phosphorylation or degradation [9,10], and increased SUMO-1 modifications of IκBα by adenosine inhibition of phosphorylation and degradation of IκBα, which attenuates NF-κB activation [11]. Our work in recent years has found that adenosine inhibits NF-κB through actions on proteasomal degradation of IκB proteins via an alternative adenosine-mediated mechanism. Studies using an NF-κB luciferase reporter assay confirmed that adenosine, generated under the adaptive pathway of hypoxic preconditioning, significantly suppressed NF-κB activation [12]. As previous studies had demonstrated a connection between IκB degradation, NF-κB inhibition and Cullin-1 (Cul-1) protein deneddylation [13,14], the neddylation status of Cul-1 after AR stimulation revealed that adenosine indeed modulated Cul-1 neddylation and further influenced IκB protein stabilization and downstream targets. Regulated protein degradation is an essential part of cell signaling for many adaptive processes. The proteasomal degradation of IκB (Fig. 1), which inhibits NF-κB, is but one example of a rapid response by the cell to signal for growth, differentiation, apoptosis, or inflammation. The E3 SCF ubiquitin ligase is specific to the IκB family and is comprised of SKP1, Cul-1 and the F-box domain of β-TrCP and is responsible for the polyubiquitination of IκB [15]. The COP9 signalsome (CSN) must conjugate the small protein Nedd8 to Cul-1 in order for the E3 SCF to be active, and deneddylated Cul-1 therefore inhibits the ubiquitination of IκB, inactivating NF-κB [16]. It is currently unclear which of these adenosine-mediated pathways predominately regulates NF-κB activity.

One mechanism of deneddylation occurs through the deneddylase-1 (DEN-1, also called SENP8) protein. DEN-1 is a Nedd8-specific protease that has isopeptidase activity capable of directly deneddylating cullin targets [17,18]. The influence of these other cullin targets on pathways that can mediate inflammation, such as Cullin-2 (Cul-2) and the HIF pathway, and the potential influence of adenosine on DEN-1 activity are areas currently being studied.

## 3 Adenosine and Ischemia and Reperfusion Injury

Ischemia-reperfusion injury is a pathologic condition characterized by an initial restriction of blood supply, followed by the subsequent restoration of perfusion and concomitant re-oxygenation. In its classic manifestation, occlusion of a coronary artery is caused by a coronary thrombus and results in a severe imbalance of metabolic supply and demand causing tissue hypoxia [19]. In the second stage of the disease, blood flow is rapidly restored. Somewhat surprisingly, the restoration of blood flow along with re-oxygenation is in many instances associated with an exacerbation of tissue injury and a profound inflammatory response (so called “reperfusion injury”) [20].

A recent study provided new insights into potential mechanisms of how adenosine and HIF-1 could function in mediating cardioprotection from ischemia and reperfusion injury [21,22]. It has been known for some time that one of the critical functions of HIF is the induction of glycolytic enzymes, which are considered critical components of enhancing the capacity of hypoxic or ischemic tissues to increase anaerobic glycolysis, and concomitant ATP production during hypoxic or anoxic conditions [23,24] Interestingly, this function of HIF1A involves the interaction with the circadian rhythm protein Period 2 (Per2), a molecule which is induced by adenosine 2B receptor activation [22]. Indeed, HIF-dependent induction of the glycolytic program during myocardial ischemia is completely abolished in *Per2*^−/−^ mice [22]. Additional studies in Hif or Per2 reporter mice provided further evidence for an interaction between HIF and PER in the heart. These studies indicated that similar to the circadian alteration of Per2 levels over a 24 h our time period, Hif1a exhibits a circadian expression pattern [22,25]. Moreover, genetic ablation of *Per2* in Hif reporter mice arrested the circadian expression pattern of HIF, indicating a functional interaction between HIF1A and PER2. Interestingly, light-induced stabilization of cardiac Per2 expression also promoted increases in the expression of glycolytic enzymes, and concomitant cardio-protection, indicating that PER2/HIF1A interact during myocardial ischemia to increase the anaerobic glycolytic capacity of the ischemic heart [22].

## 4 NF-κB and the Neddylation Pathway

Understanding the mechanisms that control responses to inflammation is important in developing effective therapies. One such condition for which novel therapies are needed is sepsis. Recent work has demonstrated that CD73-derived adenosine may be beneficial in sepsis [26], and AR activation may offer a new therapeutic approach to manage sepsis [27]. The mechanism of sepsis remains incompletely understood, though loss of endothelial cell function and subsequent activation of the immune system are hallmarks [28,29]. The NF-κB pathway is important for intracellular pro-inflammatory signaling [30] and is regulated by Cullin-RING ligases, as described above (Fig. 1).

In recent work, Ehrentraut et al. sought to identify the proximal regulator that is central to the anti-inflammatory properties elicited by adenosine in previous models of inflammation [31]. The discovery of DEN-1 provided new insights into how Cul neddylation was regulated. Therefore, in this study, the role of DEN-1-mediated neddylation during inflammation was investigated using several approaches, including knockdown and overexpression of DEN-1 in human endothelial cells. Over 50% knockdown of DEN-1 message was achieved in human microvascular endothelial cells (HMEC-1), leading to an 85% loss of DEN-1 protein expression. Cul-1 neddylation was decreased in the DEN-1 knockdown cells and a decreased translocation of NF-κB subunit p65 to the nucleus was observed after stimulation with LPS in the knockdown cells. Overexpression of DEN-1 in HMEC-1 cells significantly influenced NF-κB responses as basal NF-κB reporter plasmid activity increased by 2.8 ± 0.5 fold. These loss- and gain-of-function studies clearly implicate DEN-1 as a critical component of vascular endothelial NF-κB responses.

Recently, pharmacological inhibition of cullin neddylation has been studied using a small molecular inhibitor, MLN4924. MLN4924, a structural analog of adenosine monophosphate (AMP), functions by inhibiting the Nedd8-activating enzyme (NAE), thereby inhibiting the neddylation of cullin proteins including Cul-1 and Cul-2 [32] (Fig. 1). When MLN4924 was employed in the NF-κB pathway studies, Ehrentraut et al. found that NF-κB luciferase reporter activity was decreased in HMEC-1 cells, even after stimulation with LPS. Indeed, the influence of MLN4924 on Cul-1 neddylation was confirmed by western blot as treatment of HMEC-1 cells with MLN4924 in the presence of LPS led to Cul-1 deneddylation. The function of endothelial cells was also influenced as treatment with MLN4924 decreased the expression of ICAM-1 transcript and abrogated barrier dysfunction elicited by LPS as measured by FITC-dextran flux.

In translating these in vitro findings, the influence of disruption of the neddylation pathway with regard to inflammation was studied in vivo. Genetic studies are limited given the presumed embryonic lethality of DEN-1 knockout, therefore MLN4924 was employed for these purposes. MLN4924 is well tolerated in murine tumor models [32]. Mice were pre-exposed to MLN4924 for 1 h, followed by intraperitoneal LPS for 6 h before serum was collected to analyze cytokine and chemokine levels [31]. Interestingly, pro-inflammatory cytokines, such as IL-1β, IFNγ and TNF-α were downregulated in LPS-treated mice that had received MLN4924 pre-treatment. These in vivo observations reveal a potent anti-inflammatory role for MLN4924 and parallel deneddylation of Cul-1. Taken together, these results demonstrate that inflammation actively deneddylates Cul-1, DEN-1 is central to the control of NF-κB activation, and the neddylation inhibitor MLN4924 inhibits NF-κB activity. The role of ARs in murine dextran sulfate sodium (DSS)-colitis as a model of inflammation was also previously explored and revealed the importance of the A_2B_A receptor (A_2B_AR) [33]. The severity of colitis was increased in Aa2br^(−/−)^ mice relative to Aa2br^(+/+)^ controls, as reflected by increased weight loss, colonic shortening, and disease activity indices. Additionally, Cul-1 was constitutively neddylated in the Aa2br^(−/−)^ mice compared to the controls, again providing a link between adenosine signaling, Cul-1 neddylation and the resolution of inflammation.

## 5 HIF-1α and the Neddylation Pathway

Previous work has indicated that the microenvironment of an inflammatory lesion is depleted of oxygen and can be characterized by the generation of large amounts of adenine nucleotide metabolites [34,35]. HIF proteins function as one of the master regulators of oxygen homeostasis. HIF coordinates adenine nucleotide metabolism and regulates adenosine signaling [4]. Together, hypoxia and adenosine have protective actions during inflammation, promoting resolution and tissue repair. As mentioned previously, an analogous E3 ligase complex exists upstream of the alpha subunits of HIF, which is comprised of Cul-2, Elongin B/C and the von Hippel–Lindau protein (pVHL). Similar to the Cul-1–IκB–NF-κB axis, neddylation of Cul-2 is required for the ubiquitination of hydroxylated HIF-1α in normoxic conditions (Fig. 1). The alpha subunits of HIF are hydroxylated by prolyl hydroxylases (PHDs) to target the HIF subunits for degradation. In the absence of oxygen, the alpha subunits of HIF are not targeted for degradation and therefore translocate into the nucleus to dimerize with constitutively expressed HIF-1β subunits, turning on targeting genes [4]. PHD inhibitors have been previously investigated for their therapeutic potential in various disease models, such as murine colitis [36,37].

In our recent work, an alternative mechanism of HIF-1α stabilization via the Cul-2 neddylation pathway was explored in intestinal epithelial cells [38]. Given the previous results regarding inhibition of the NF-κB pathway after Cul-1 deneddylation both genetically through DEN-1 knockdown and pharmacologically through MLN4924 treatment, we hypothesized that stabilization of HIF-1α by Cul-2 deneddylation would be protective in mucosal inflammation. Indeed, studies in HeLa cells as models of epithelial cells revealed robust HIF-1α stabilization and Cul-2 deneddylation after acute treatments with low concentrations of MLN4924. The functional consequences of this HIF-1α stabilization were investigated. HIF-1α activity significantly increased after treatment with MLN4924 as measured by an HRE (hypoxia response element) luciferase reporter assay, and the HIF-1α target genes BNIP3L and enolase-1 were significantly upregulated 1 and 2 h after treatment with MLN4924.

The knockdown of DEN-1 in T84 cells, an intestinal epithelial cell line, via lentivirus shRNA revealed decreased Cul-2 neddylation at baseline, increased barrier formation over time and an increased rate of resolution after a calcium switch assay, compared to cells infected with a non-targeting control lentivirus shRNA. The protective role of HIF and the contribution of the neddylation pathway were further confirmed in a DSS-colitis murine model. Mice treated with DSS that received a pre-treatment of MLN4924 displayed decreased percent body weight loss, decreased colon shortening and decreased histologic injury scores, compared to mice receiving DSS and vehicle treatment. These results suggest that modulation of the neddylation pathway via MLN4924 under inflammatory conditions can be protective when HIF stabilization is promoted. Interestingly, HIF hydroxylases can also regulate NF-κB, and NF-κB is thought to be protective in the intestinal epithelium via the inhibition of enterocyte apoptosis [36,39,40]. Therefore, the ability of MLN4924 to promote HIF signaling and inhibit NF-κB signaling should be carefully balanced for the neddylation pathway to represent a novel therapeutic target for inflammatory bowel diseases. Studies to address these concepts are currently ongoing.

## 6 Conclusion

The interplay of molecular signaling that directs inflammation represents a complex system. Understanding the crosstalk between these signals and how they might be modulated to treat inflammation-related diseases is an important area of research. Adenosine is a molecule that exerts broad biological actions and new studies have shed light on its influence in pivotal inflammatory pathways, including NF-κB and HIF. Using the mechanisms of post-receptor adenosine signaling that have been recently discovered, new potential therapeutic targets have been identified for conditions such as sepsis and IBD that warrant additional study.

## Conflict of Interest

The authors declare no financial interests in any of the work submitted here.
